# Patient-perceived recovery after posterior spinal fusion: evaluating minimum clinically important difference (MCID) in adolescents with idiopathic scoliosis

**DOI:** 10.1007/s43390-026-01374-2

**Published:** 2026-05-02

**Authors:** Leila Mehraban Alvandi, J. Nicholas Charla, Zachariah Samuel, Edina Gjonbalaj, Mohamed Said, Jorden Xavier, Carolyn Rachofsky, Morgan Roche, Priya Singh, Yungtai Lo, Jacob Schulz, Jaime A. Gomez, Eric D. Fornari

**Affiliations:** 1 Division of Pediatric Orthopedic Surgery, Department of Orthopaedic Surgery, Montefiore-Einstein, Bronx, New York USA; 2 Albert Einstein College of Medicine, Bronx, New York USA

**Keywords:** Adolescent idiopathic scoliosis (AIS), Posterior spinal fusion (PSF), Patient-reported outcomes (PROs), Scoliosis research society-22r (SRS-22r), Minimum clinically important difference (MCID), Mental health, Pain, Self-image, Longitudinal outcomes

## Abstract

**Background:**

Adolescent idiopathic scoliosis (AIS) affects 1–3% of children in the United States, with approximately 38,000 patients undergoing posterior spinal fusion (PSF) annually. The relationship between preoperative patient-reported outcomes, postoperative recovery, and long-term clinical significance remains unclear. This study assesses longitudinal changes in Scoliosis Research Society-22r (SRS-22r) scores. It evaluates clinical significance using the Minimum Clinically Important Difference (MCID) in AIS patients undergoing PSF.

**Study design:**

Retrospective study using prospectively collected data.

**Methods:**

A retrospective study was conducted using prospectively collected data on AIS patients who underwent PSF at a single academic institution between 2012 and 2022. Patient-reported outcomes were assessed using the SRS-22r questionnaire at preoperative, 6-month, 1-year, and ≥ 2 years postoperative time points. MCID threshold achievements were determined using anchor-based criteria from Bago et al. The percentage of patients achieving MCID and predictors of MCID achievement were analyzed using logistic regression.

**Results:**

A total of 161 patients (mean age 15.26 ± 2.15 years; 65.8% female) were included. At 1 year, MCID achievement ranged from 30.1% (Self-Image) to 43.4% (Mental Health). By ≥ 2 years, MCID rates declined in Pain (25.9%) and Self-Image (22.8%) but increased in Function/Activity (44.1%). Lower preoperative SRS-22r scores consistently predicted MCID achievement across all domains. A documented mental-health history reduced the likelihood of Pain MCID at 1 year, and larger postoperative Cobb angles independently decreased the odds of achieving Self-Image MCID at both follow-up points. Neighborhood opportunity (Child Opportunity Index) was not associated with outcomes. Sensitivity analyses demonstrated that complete-case ≥ 2 year MCID rates were consistently bounded by best- and worst-case values and closely approximated LOCF estimates, supporting robustness despite attrition.

**Conclusion:**

Meaningful postoperative improvement after PSF varies substantially by SRS-22r domain. Pain and mental-health gains occurred early and stabilized, whereas function demonstrated ongoing recovery, and self-image improved rapidly and remained stable. MCID achievement was most likely in patients with greater preoperative symptom burden, while mental-health history and residual postoperative deformity diminished domain-specific improvements. The stability of MCID patterns across sensitivity analyses reinforces the reliability of long-term findings. These results highlight the importance of incorporating psychological assessment, expectation management, and attention to postoperative alignment into perioperative care for AIS patients.

## Introduction

Adolescent idiopathic scoliosis (AIS) affects 1–3% of children in the United States aged 10–18 [1, 2]. Most patients with AIS experience a degree of postoperative back pain following posterior spinal fusion (PSF) due to the invasive nature of the procedure [3]. While this pain often improves with recovery, some patients report long-lasting worsening pain, affecting function and quality of life [4–7].

Prior studies have suggested that psychosocial factors may contribute to patients with AIS experiencing greater degrees of postoperative pain following PSF [8, 9]. Studies show that pain catastrophizing, anxiety, and preoperative depression are linked to postoperative chronic pain [8–12].

The Scoliosis Research Society-22r (SRS-22r) questionnaire evaluates patient-reported outcomes (PROs) in patients with AIS undergoing PSF. The Minimum Clinically Important Difference (MCID) represents the smallest meaningful improvement in health status. MCID has been utilized in clinical practice to provide clinical meaning to changes in outcome measures. It measures whether a patient feels improved compared to the variation in outcome scores [13–15]. While preoperative SRS-22r scores may impact postoperative outcomes, their predictive value for MCID achievement remains unclear at longer follow-up time points. Few studies have assessed longitudinal preoperative and postoperative SRS-22r scores and MCID thresholds to interpret changes in mental health, self-image, and quality-of-life outcomes at 1 year and ≥ 2 years follow-up after PSF [16–30].

This study aims to assess longitudinal changes in SRS-22r scores in AIS patients undergoing PSF and determine the clinical significance of these changes using anchor-based MCID thresholds [15, 26, 27]. By classifying patients as achieving or not achieving MCID across the pain, function and activity, self-image, and mental health domains, this study aims to provide a clinically meaningful assessment of postoperative recovery. Moreover, this study examines predictors of MCID achievement and identifies the main preoperative and demographic factors that may influence long-term PROs following surgery.

## Materials and methods

A prospectively collected database of AIS patients treated with PSF (with a minimum 2-year follow-up) at a single academic institution between January 2012 and December 2022 was retrospectively reviewed to obtain demographic and clinical data. Institutional Review Board approval was obtained prior to initiating the study. Exclusion criteria included patients aged > 21 at the time of surgery, those with non-idiopathic diagnoses, and those who underwent alternative surgical procedures such as anterior spinal fusion. No limitations were placed on Cobb angles or the number of levels fused.

PROs were measured using the SRS-22r questionnaire at preoperative and at 6 week, 6 month, 1 year, and ≥ 2 year postoperative time points. It assesses pain, function and activity, self-image, mental health, and satisfaction (scored 1–5 per item; domain scores range from 5 to 25, and satisfaction scores range from 2 to 10) [26].

Neighborhood socioeconomic context was quantified using the Child Opportunity Index (COI), a percentile-based metric (0–100) linked to patient ZIP code. COI was analyzed as a continuous variable (scaled per 10-percentile increase) to preserve statistical power and avoid information loss associated with categorization. For descriptive clarity, we also report the distribution of COI categories (Very Low/Low vs. Moderate/High/Very High). Continuous variables were summarized using means ± standard deviations (SD) or medians with interquartile ranges (IQRs). Categorical variables were described using counts and percentages.

### Minimum clinically important difference (MCID) and missing data strategy

This study used anchor-based MCID thresholds established by Bago et al. for the SRS-22r domains: 0.6 for Pain, 0.3 for Function and Activity, 1.3 for Self-Image, and 0.3 for Mental Health; the Satisfaction domain was excluded from MCID calculations [25–27]. The primary responder analysis defined MCID achievement as meeting or exceeding these thresholds between the preoperative assessment and the 1 year or ≥ 2 year postoperative assessments.

Given the potential influence of loss to follow-up, MCID responder analyses were structured in two stages. First, complete-case analyses were performed using denominators restricted to patients with available scores at baseline, 1 year, and ≥ 2 years for the domain of interest. Second, to assess the robustness of ≥ 2 year MCID findings to missing data, three complementary sensitivity approaches were applied for each domain:

1. Best-case: all missing ≥ 2 year scores assumed responders;
2. Worst-case: all missing ≥ 2 year scores assumed non-responders;
3. Last observation carried forward (LOCF): 1 year MCID status was carried forward when the ≥ 2 year score was missing; patients missing both 1 year and ≥ 2 year data remained missing.

This framework bounded ≥ 2 year MCID rates across plausible assumptions and minimized bias due to attrition.

### Longitudinal change in SRS-22r Scores

Within-subject changes in SRS-22r domain scores (Pain, Mental Health, Function, and Self-Image) were evaluated at four time points: Pre-Op, 6 months, 1 year, and ≥ 2 years. Because follow-up was unbalanced across patients and score distributions were non-normal, nonparametric methods were used. Overall, within-domain change was assessed using the Friedman test. When significant, pairwise comparisons were conducted using Wilcoxon signed-rank tests with Holm correction for multiple comparisons. Mean ± SD values and sample sizes were plotted to illustrate longitudinal recovery trajectories.

### Predictors of MCID achievement

For each domain, separate logistic regression models examined predictors of MCID achievement at 1 year and ≥ 2 years. Baseline domain score was forced into all models, and additional covariates (e.g., sex, age, mental health history, post-op Cobb angle) were limited to maintain an events-per-variable (EPV) ratio ≥ 10. 28 When EPV < 10, results were interpreted as exploratory. Adjusted odds ratios (ORs) with 95% confidence intervals (CI) were reported.

Statistically significant differences were defined as P < 0.05. All analyses used SPSS v.24 (IBM SPSS Statistics for Windows, Version 24.0, Armonk, NY, 2016).

## Results

**Fig. 1:**
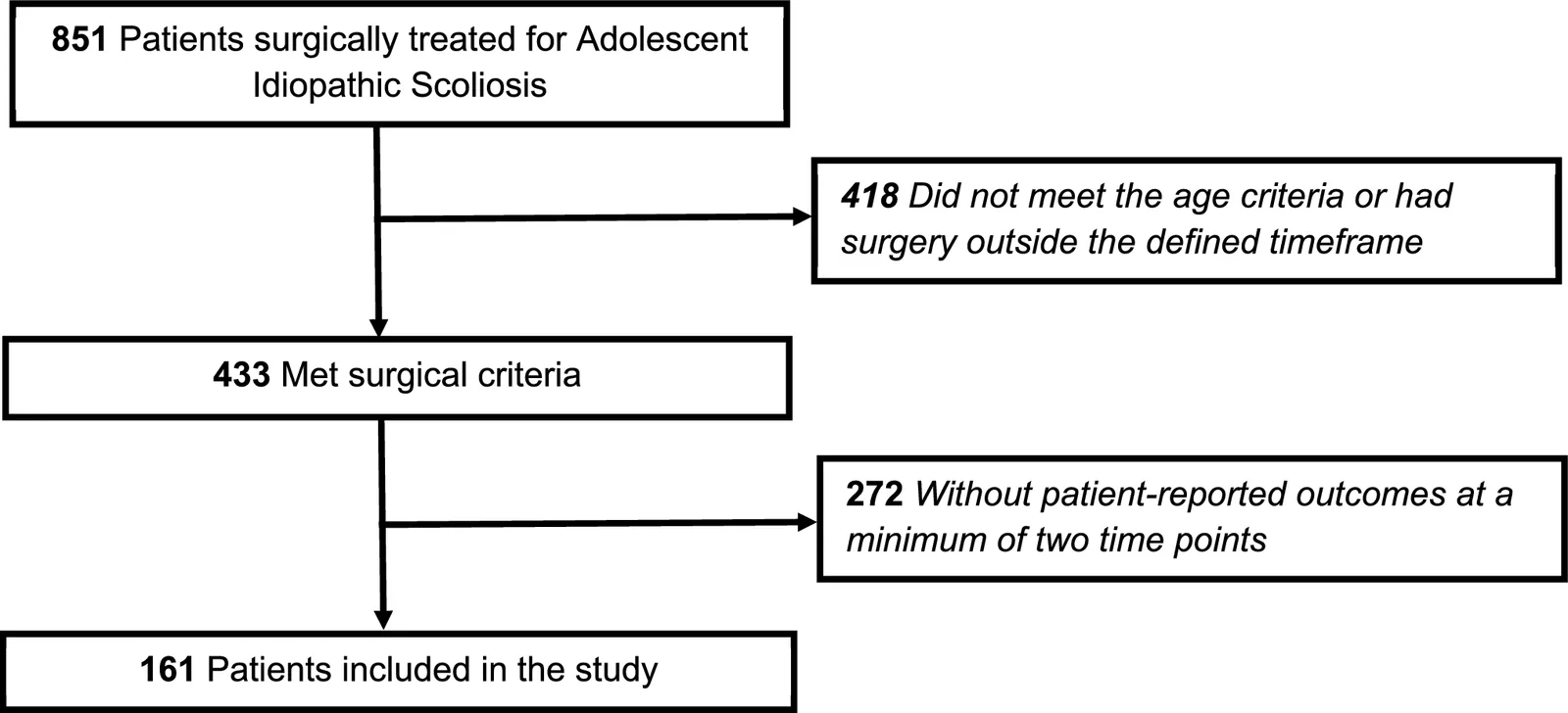
Flowchart illustrating the selection criteria for inclusion. A total of 851 patients underwent surgical treatment for adolescent idiopathic scoliosis. Of these, 418 patients did not meet the age criteria or had surgery outside the defined timeframe, leaving 433 patients who met the criteria. After excluding 272 who were missing preoperative and postoperative patient-reported outcome (PRO) data at a minimum of two time points, 161 patients remained for the final analysis

After applying exclusion criteria (Fig. 1), 161 patients were included with a mean age of 15.26 ± 2.15 years. Patient demographics and characteristics are summarized in Table 1. The majority were female (65.8%). The racial distribution included 5.0% White, 38.5% Black, and 41.6% categorized as "Other," with 14.9% of the race data unavailable. Hispanic ethnicity was reported in 34.2% of participants, while 39.8% were non-Hispanic, and ethnicity was unavailable for 26.1%. According to the BMI classification, 6.2% were underweight, 57.8% had a normal weight, 19.3% were overweight, and 16.8% were obese. Neighborhood opportunity demonstrated substantial variability across the cohort, with a mean COI (per 10-percentile scale) of 2.39 ± 2.53 (range 0.1–9.8). The majority (78.9%) resided in areas classified as very low or low for overall COI. A mental health history was documented in 14.9% of participants. The median preoperative Cobb angle was 56.0°, with a range of 43.0 to 96.0°. The mean postoperative Cobb angle was 17.36 ± 7.70. The median number of spinal levels fused was 12 (5–15). The median length of the hospital stay was 4 days, ranging from 2 to 51 days. One patient had a 51 day stay due to a complication of the Superior Mesenteric Artery (SMA) Syndrome.

**Table 1:**
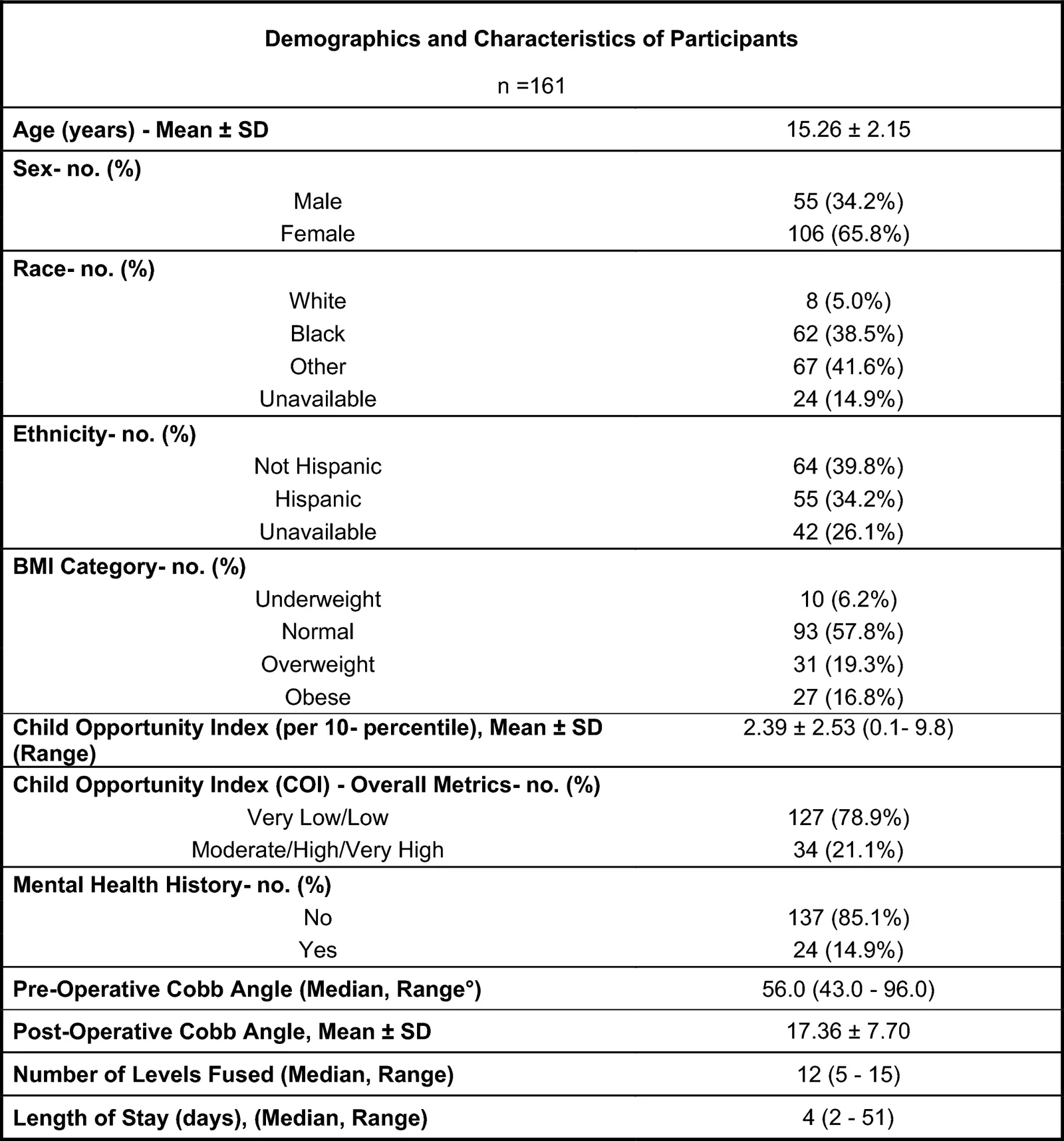
Patient demographics and characteristics

### MCID achievement analysis across SRS-22r domains

Table 2 presents the number and percentage of patients who achieved and did not achieve anchor-based MCID thresholds across the SRS-22r domains at 1 year and ≥ 2 years postoperative follow-ups, based on Bago et al.'s anchor-based MCID criteria [26, 27]. At 1 year, MCID achievement was observed in 43.4% of patients for Mental Health, 34.6% for Pain, 36.1% for Function and Activity, and 30.1% for Self-Image. At ≥ 2 years, complete-case MCID rates were 35.0% for Mental Health, 25.9% for Pain, 44.1% for Function and Activity, and 22.8% for Self-Image.

**Table 2:** Number and percentage of patients who did and did not pass Bago et al. [25, 26] ‘s anchor-based MCID thresholds (Across SRS-22r Domains) (A indicates anchor based)

SRS22r domains	MCID achievement status n (%)	MCID threshold
Mental Health (1 year postoperative)		0.3
Not achieved	47 (56.6)	
Achieved	36 (43.4)	
Mental Health (≥ 2 years postoperative)		0.3
Not achieved	39 (65.0)	
Achieved	21 (35.0)	
Pain (1 Year postoperative)		0.6
Not achieved	53 (65.4)	
Achieved	28 (34.6)	
Pain (≥ 2 years postoperative)		0.6
Not achieved	63 (74.1)	
Achieved	22 (25.9)	
Function and activity (1 year postoperative)		0.3
Not achieved	53 (63.9)	
Achieved	30 (36.1)	
Function and activity (≥ 2 years postoperative)		0.3
Not achieved	33 (55.9)	
Achieved	26 (44.1)	
Self-image (1 year postoperative)		1.3
Not achieved	58 (68.9)	
Achieved	25 (30.1)	
Self-image (≥ 2 years postoperative)		1.3
Not achieved	44 (77.2)	
Achieved	13 (22.8)	

### Sensitivity analyses for ≥ 2-year MCID

Sensitivity analyses were conducted to assess the effect of missing ≥ 2 year data on MCID estimates. Complete-case responder rates ranged from 32.3 to 54.8% across domains. LOCF estimates were similar to complete-case values for Pain (35.0% vs. 34.1%) and Self-Image (32.3% for both), while larger differences were observed for Mental Health and Function due to higher proportions of missing data. Under best-case assumptions, MCID rates increased to 60.9%–79.5%, whereas worst-case assumptions produced lower rates (8.1%–16.1%). Across all domains, the best-case and worst-case values consistently bounded the complete-case estimates, supporting the stability of the ≥ 2 year MCID patterns despite attrition (Table 3).

**Table 3:** Sensitivity analyses for ≥ 2-year MCID responder rates across SRS-22r domains

Domain	Complete-case n/N (%)	LOCF n/N (%)	Best-case n/N (%)	Worst-case n/N (%)
Pain	14/41 (34.1)	14/40 (35.0)	98/161 (60.9%)	22/161 (13.7%)
Mental health	16/31 (51.6)	36/83 (43.4)	122/161 (75.8%)	21/161 (13.0%)
Function	17/31 (54.8)	52/161 (32.3)	128/161 (79.5%)	26/161 (16.1%)
Self-image	10/31 (32.3)	52/161 (32.3)	117/161 (72.7%)	13/161 (8.1%)

### Mean SRS-22r domain scores for MCID achievement

Table 4 presents the mean SRS-22r domain scores for patients who did and did not achieve MCID, both preoperatively and at ≥ 2 years postoperatively, with corresponding p-values indicating statistical significance. Patients who achieved MCID had significantly lower preoperative scores across the mental health, pain, function and activity, and self-image domains. Conversely, at ≥ 2 years postoperatively, these patients demonstrated significantly higher scores across the exact domains, reflecting sustained clinical improvement. Overall, lower preoperative scores were associated with MCID achievement.

**Table 4:** Mean SRS-22r Domain Scores for Patients Passing vs. Not Passing MCID Threshold

	Mean	*P* value
**Mental Health (Preoperative)**		**<0.001**
Not Achieved	4.11 ± 0.68	
Achieved	3.41 ± 0.71	
**Mental Health (≥ 2 Years Postoperative)**		**0.022**
Not Achieved	3.72 ± 0.91	
Achieved	4.25 ± 0.66	
**Pain (Preoperative)**		**0.045**
Not Achieved	4.06 ± 0.75	
Achieved	3.67 ± 0.83	
**Pain (≥ 2 Years Postoperative)**		**0.01**
Not Achieved	3.97 ± 0.86	
Achieved	4.30 ± 0.59	
**Function a**nd** Activity (Preoperative)**		**<0.019**
Not Achieved	4.06 ± 0.55	
Achieved	3.68 ± 0.62	
**Function a**nd** Activity (≥ 2 Years Postoperative)**		**<0.001**
Not Achieved	3.59 ± 0.68	
Achieved	4.50 ± 0.43	
**Self-Image (Preoperative)**		**<0.001**
Not Achieved	3.44 ± 0.60	
Achieved	2.66 ± 0.72	
**Self-Image (≥ 2 Years Postoperative)**		**<0.001**
Not Achieved	3.79 ± 0.76	
Achieved	4.55 ± 0.45	

### Longitudinal recovery patterns across SRS-22r domains

**Fig. 2:**
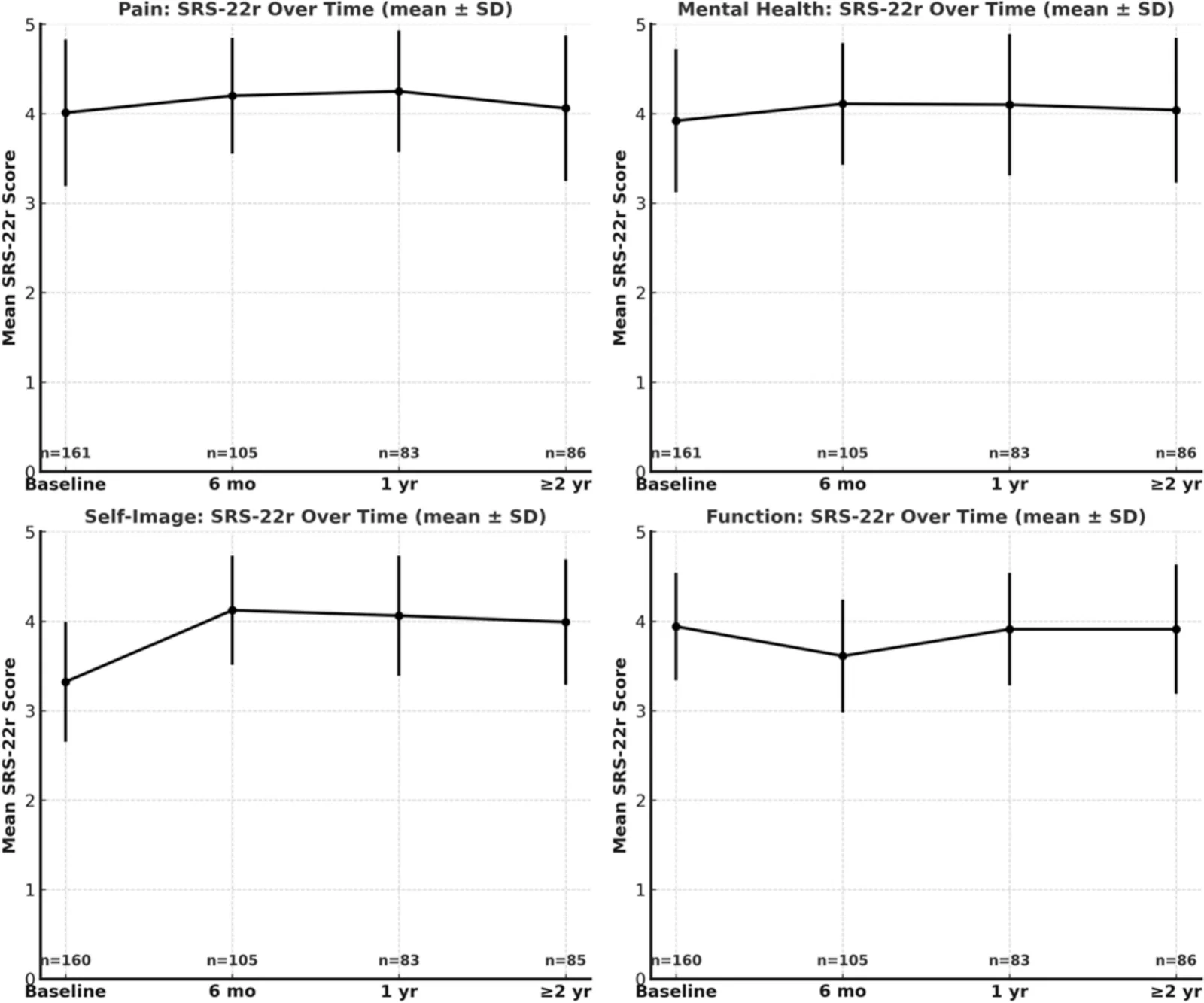
Longitudinal trajectories of SRS-22r domain scores over time (mean ± SD)

A four-panel line graph displays mean ± SD and sample sizes at each time point for all SRS-22r domains (Fig. 2). **Pain** improved significantly from Pre-Op to 6 months (P = 0.018) and from Pre-Op to 1 year (P = 0.007), with no additional changes beyond 1 year (all P ≥ 0.219). After Holm correction, only the Pre-Op to 1-year comparison remained significant, indicating that pain relief peaks within the first postoperative year and then stabilizes. **Mental Health** showed modest improvement from Pre-Op to 6 months (P = 0.024) and to 1 year (P = 0.011), although neither comparison remained significant following Holm correction. No additional changes occurred beyond 1 year, suggesting early psychological gains followed by plateau. **Function/Activity** declined significantly at 6 months (P < 0.001) but improved markedly at 1 year (P < 0.001) and ≥ 2 years (P < 0.001) relative to the 6-month time point. Additional gains were observed between 1 year and ≥ 2 years (P = 0.010). All significant findings persisted after Holm correction, reflecting an initial postoperative decline followed by progressive and sustained functional recovery. **Self-Image** improved substantially from Pre-Op to every postoperative interval (all P < 0.001; r = 0.65–0.75). No significant differences were observed among postoperative time points (all P ≥ 0.358), indicating early and stable enhancement in appearance-related satisfaction following surgical correction. Across domains, the most significant improvements occurred between 6 and 12 months, with no evidence of decline (“drop-off”) at ≥ 2 years.

### Predictors of MCID achievement

Across all SRS-22r domains, lower preoperative subdomain scores were the most consistent predictors of achieving MCID, suggesting greater postoperative improvement potential. After applying EPV-constrained model selection, demographic and socioeconomic variables, including sex, BMI, and COI, were generally not associated with MCID achievement (Table 5).

**Table 5:** Significant predictors of SRS-22r MCID achievement after posterior spinal fusion

Domain & timepoint	Predictor	Adjusted OR	95% CI	P-value	EPV	Interpretation
Pain–1 year	Baseline pain	0.07	0.02–0.24	** < 0.001**	14	Significant
	Mental-health history	0.11	0.01–0.80	**0.030**	14	Significant
Pain – ≥ 2 years	Baseline pain	0.47	0.24–0.89	**0.021**	11	Significant
	Male sex	2.93	0.98–8.75	0.054	11	Trend
Mental health – 1 year	Baseline mental health	0.21	0.10–0.45	** <0 .001**	13	Significant
Mental health – ≥ 2 years	Baseline mental health	0.25	0.11–0.61	**0.002**	11	Significant
Self-image – 1 year	Baseline self-image	0.07	0.02–0.26	** < 0.001**	12	Significant
	Postoperative Cobb angle	0.89	0.81–0.98	**0.019**	12	Significant
Self-Image – ≥ 2 years	Baseline self-image	0.05	0.01–0.34	**0.002**	**6.5**	Exploratory
	Postoperative Cobb angle	0.83	0.71–0.97	**0.022**	**6.5**	Exploratory
Function/activity – 1 year	Baseline function/activity	0.10	0.03–0.28	** < 0.001**	15	Significant
Function/activity – ≥ 2 years	Baseline function/activity	0.34	0.13–0.88	**0.027**	12	Significant

### Pain

Lower baseline Pain was associated with higher odds of achieving MCID at both 1 year (aOR = 0.07, 95% CI 0.02–0.24, P < 0.001) and ≥ 2 years (aOR = 0.47, 95% CI 0.24–0.89, P = 0.021). A history of mental-health disorder was associated with reduced odds of 1-year Pain MCID (aOR = 0.11, 95% CI 0.01–0.80, P = 0.030). Male sex showed a borderline association with ≥ 2-year Pain MCID (P = 0.054) but did not reach statistical significance.

### Mental health

Higher baseline Mental Health scores predicted lower odds of MCID achievement at both 1 year (aOR = 0.21, 95% CI 0.10–0.45, P < 0.001) and ≥ 2 years (aOR = 0.25, 95% CI 0.11–0.61, P = 0.002). Neither sex nor mental-health history was significant in the final models.

### Self-image

At 1 year, higher baseline Self-Image substantially reduced the likelihood of achieving MCID (aOR = 0.07, 95% CI 0.02–0.26, P < 0.001), while larger postoperative Cobb angle independently predicted lower odds of MCID (aOR = 0.89, 95% CI 0.81–0.98, P = 0.019), indicating an ~ 11% decrease in the probability of clinically meaningful improvement in appearance for each additional degree of residual deformity. This 1-year model is interpreted as exploratory, given the limited number of events (EPV ≈ 7.5). At ≥ 2 years, higher baseline Self-Image (aOR = 0.05, 95% CI 0.01–0.34, P = 0.002) and larger postoperative Cobb angle (aOR = 0.83, 95% CI 0.71–0.97, P = 0.022) again independently predicted reduced odds of MCID achievement. These ≥ 2 year findings are described as exploratory due to the small number of events.

### Function/activity

Lower baseline Function/Activity scores predicted higher odds of MCID achievement at both 1 year (aOR = 0.10, 95% CI 0.03–0.28, P < 0.001) and ≥ 2 years (aOR = 0.34, 95% CI 0.13–0.88, P = 0.027). Sex was not associated with MCID in either model.

## Discussion

This study evaluated meaningful postoperative recovery after PSF for AIS using validated anchor-based MCID thresholds across all SRS-22r domains [16, 25–27, 29]. While mean domain scores showed early postoperative improvement and long-term stability, MCID achievement varied across domains. This distinction is clinically important: MCID reflects within-patient meaningful change, and lower rates at ≥ 2 years likely reflect a plateau in postoperative gains rather than true deterioration. Similar domain-specific variability and ceiling effects have been described in prior AIS studies, underscoring the importance of MCID-based interpretation [26, 27], Longitudinal recovery patterns further clarify the timing of improvement: Pain, Mental Health, and Self-Image demonstrated their largest gains within the first 6 to 12 months and then plateaued without evidence of decline at ≥ 2 years. Function followed the expected pattern of early postoperative limitation with recovery by 1 year and continued improvement thereafter. Overall, most AIS patients reached near-maximal recovery within the first postoperative year, and despite reduced follow-up at later time points, no drop-off in outcomes was observed among available respondents.

Across domains, lower preoperative SRS-22r scores consistently predicted MCID achievement, suggesting that patients with more significant preoperative symptoms showed the greatest postoperative gains. This pattern aligns with broader PRO literature, in which ceiling effects restrict the magnitude of improvement among patients with high preoperative functioning [27]. In the Pain domain, lower preoperative pain was associated with higher odds of MCID achievement at both 1 year and ≥ 2 years, while a documented mental-health history reduced the likelihood of clinically meaningful pain improvement. Male sex demonstrated a nonsignificant trend toward higher odds of Pain MCID at ≥ 2 years; this finding should be interpreted cautiously but aligns with literature describing sex-based differences in pain perception and recovery trajectories [30–38]. Collectively, these findings highlight the influence of psychological factors on postoperative pain and support the importance of early psychosocial assessment and perioperative mental-health support.

For Self-Image, higher baseline Self-Image scores and larger postoperative Cobb angles were associated with lower MCID achievement. These results emphasize the relevance of residual deformity in shaping appearance-based satisfaction and the value of realistic preoperative counseling regarding expected cosmetic outcomes. Functional recovery followed a typical postoperative trajectory, early decline followed by gradual improvement, and was primarily influenced by baseline functioning rather than demographic or socioeconomic factors [16, 37–51].

Sensitivity analyses using best-case, worst-case, and LOCF assumptions demonstrated that ≥ 2 year MCID patterns remained stable across missing-data scenarios. These findings strengthen confidence in the robustness of the observed associations and address a standard limitation of AIS outcomes research: variation in long-term follow-up rates.

Overall, this study identifies preoperative symptom severity, mental-health history, and residual postoperative deformity as key determinants of clinically meaningful improvement after PSF for AIS. MCID-based interpretation provides insights beyond mean-score analysis by identifying which patients are most likely to benefit in each SRS-22r domain and where additional clinical support may be needed.

## Limitations

This single-institution study may limit generalizability, and loss to follow-up at ≥ 2 years introduces potential attrition bias despite robust sensitivity analyses. In addition, the use of anchor-based MCID thresholds has inherent limitations. MCID values are derived from external cohorts and may have limited discriminative accuracy, which can affect the ability to distinguish true clinical improvement from measurement variability at the individual patient level. This may result in some misclassification of patients as responders or non-responders. Furthermore, MCID is dependent on baseline status, such that patients with higher preoperative SRS-22r scores may have less capacity to achieve threshold-based improvement despite experiencing clinically meaningful benefit (ceiling effects). Although sex-based trends were observed in pain MCID, the mechanisms underlying these differences remain unexplored and warrant further investigation. Finally, psychosocial variables beyond documented mental-health history (e.g., coping style, anxiety severity) were not available but may further influence postoperative outcomes.

## Conclusion

Using validated anchor-based MCID thresholds, this study provides a patient-centered assessment of meaningful recovery after PSF for AIS. Lower preoperative symptom burden consistently predicted MCID achievement across domains, while mental-health history influenced pain improvement, and postoperative Cobb angle contributed to appearance-related outcomes. These findings underscore the importance of psychological assessment, expectation setting, and attention to residual alignment in optimizing postoperative care.

This work adds to the AIS literature by applying validated MCID thresholds across all SRS-22r domains at both 1- and ≥ 2-year follow-up and by incorporating best-case, worst-case, and LOCF sensitivity analyses to address missing data. The identification of postoperative Cobb angle as a predictor of Self-Image MCID links radiographic correction to patient-perceived appearance. At the same time, the influence of mental-health history emphasizes the role of psychosocial factors in recovery. Together, these results clarify which patients are most likely to achieve meaningful benefit and provide actionable guidance for clinical counseling, shared decision-making, and future research.

## Author contibutions

Leila Mehraban Alvandi, PhD, made substantial contributions to the study design, data acquisition, analysis, and interpretation. She drafted and critically revised the manuscript, gave final approval of the version to be published, and agreed to be accountable for all aspects of the work to ensure integrity and accuracy. Nicholas Charla and Zachariah Samuel served as co–senior authors. They made substantial contributions to the study design, data analysis and interpretation, manuscript drafting, and revision. Both gave final approval of the version to be published and agreed to be accountable for all aspects of the work in ensuring its accuracy and integrity. Priya Singh, Edina Gjonbalaj, *Mohamed Said, BS*, Carolyn Rachofsky, Morgan Roche, and Jorden Xavier contributed substantially to the study design, data acquisition and interpretation, and manuscript preparation. Each approved the final version of the manuscript and agreed to be accountable for its accuracy and integrity. Yungtai *Lo*, PhD, served as the study biostatistician. He made significant contributions to statistical design, data analysis, and interpretation. He reviewed and approved the final manuscript and agrees to be accountable for the accuracy and integrity of the statistical content. Jacob F. Schulz, MD, made substantial contributions to the conception and interpretation of data. He revised the manuscript critically for important intellectual content, approved the final version, and agreed to be accountable for all aspects of the work. Jaime A. Gomez, MD, contributed to the study design, data acquisition and interpretation, and manuscript development. He approved the final version and agreed to be accountable for the integrity of the work. Eric D. Fornari, MD, made substantial contributions to the study’s conception and data interpretation. He critically revised the manuscript, approved the final version, and agreed to be accountable for all aspects of the work.
